# Knockdown of *Tcirg1* inhibits large-osteoclast generation by down-regulating NFATc1 and IP3R2 expression

**DOI:** 10.1371/journal.pone.0237354

**Published:** 2020-08-13

**Authors:** Dongyan Zhang, Liying Lin, Bingwu Yang, Zhen Meng, Bin Zhang

**Affiliations:** 1 Department of Oral and Maxillofacial Surgery, School and Hospital of Stomatology, Shandong University & Shandong Provincial Key Laboratory of Oral Tissue Regeneration & Shandong Engineering Laboratory for Dental Materials and Oral Tissue Regeneration, Jinan, Shandong, PR China; 2 Department of Stomatology & Key Laboratory of Oral Maxillofacial-Head and Neck Medical Biology of Shandong Province & Precision Biomedical Key Laboratory, Liaocheng People’s Hospital, Liaocheng, Shandong, PR China; Charles P. Darby Children's Research Institute, UNITED STATES

## Abstract

The *TCIRG1* gene encodes the a3 isoform of vacuolar H+-ATPase (V-ATPase), which forms a proton transport channel in osteoclasts. Defects in this gene lead to functional impairment of osteoclasts and increased bone mass; however, the molecular mechanisms of *TCIRG1* loss have not been fully elucidated. In the current study, we transfected mouse bone marrow-derived monocytes with control or *Tcirg1*-knockdown lentiviruses to further investigate the mechanisms of *TCIRG1*. Our results demonstrate that knockdown of *Tcirg1* inhibits large-osteoclast (>100 μm) generation by decreasing the expression of nuclear factor of activated T-cells 1 (NFATc1) and inositol-1,4,5-trisphosphate receptor 2 (IP3R2). The decreased IP3R2 reduces intracellular calcium levels, which limits the nuclear translocation of NFATc1 in RANKL-induced mouse bone marrow-derived monocytes. These findings provide a mechanism to explain the effects of *TCIRG1* impairment, with potential implications for the development of therapies for osteopetrosis.

## Introduction

Osteoclasts are derived from mononuclear progenitors of pluripotent hematopoietic stem cells. Their main function relates to the resorption of mineralized tissues, such as bone. Osteoclasts are critical for the maintenance, repair and remodeling of bones, and any defect in osteoclasts would lead to an increase in bone mass [1]. Hereditary or acquired defects in osteoclast genesis may lead to osteopetrosis and other debilitating diseases for which there are a lack of effective therapies [2]. Therefore, an increased understanding of mechanisms of osteogenesis is essential.

V-ATPase is a highly conserved enzyme complex that is important for osteoclast function [3]. It is comprised of the proton translocation domain V_0_, which contains a, c, c', d and e subunits, as well as the ATP hydrolysis domain V_1_, which contains A-H subunits. The V_0_ domain forms a proton transport channel and has four isoforms: a1-4 [4, 5]. The a3 isoform is mainly expressed in osteoclasts and is encoded by the *TCIRG1 (Atp6i)* gene [6]. More than 50% of human malignant infantile osteopetrosis is accounted for by *TCIRG1* mutations. Furthermore, *Tcirg1*^*-/-*^ mice have significant osteopetrosis and die within 5 weeks [7, 8]. Li et al. observed that newborn *Atp6i*^*-/-*^ mice have more osteoclasts accumulated but less bone resorption [6], which is suggestive of defective bone remodeling.

The Nuclear factor of activated T-cells (NFAT), which was initially found in activated T-cells, is involved in the differentiation and function of diverse cells. The NFAT family includes NFATc1, NFATc2, NFATc3, NFATc4, and NFATc5 [9, 10]. NFATc1 is highly expressed in RANKL-induced osteoclasts. RANKL and ITAM-associated immunoglobulin-like receptor cooperate to activate phospholipase C γ (PLCγ) to produce IP3 and act on the IP3R in the endoplasmic reticulum (ER), triggering Ca^2+^ release. Subsequently, calcineurin causes the dephosphorylation of the serine residues in NFATc1, which then translocates into the nucleus to initiate osteoclastogenesis [11]. Thus, NFATc1 is an additional protein that contributes to osteoclast formation through IP3R activation.

In this study, we found that the number of osteoclasts generated by *Tcirg1* knockdown bone marrow-derived monocytes (BMMs) was increased, but the volume of these osteoclasts was smaller than in wild-type mice. Therefore, we hypothesize that knockdown of the *Tcirg1* gene may inhibit calcium oscillation by reducing the expression of IP3R, thereby limiting the nuclear translocation of NFATc1 and inhibiting large-osteoclasts generation.

## Materials and methods

### Cell culture and differentiation

All animal procedures in this study complied with the national guidelines in China, and this work was approved by the Ethics Committee of Liaocheng People’s Hospital affiliated with Shandong University (NO. 2017009). All efforts were made to reduce animal suffering.

Four to six-week old male mice were deeply anaesthetized with isoflurane prior to decapitation. Bone marrow cells were obtained from the femur and humerus and cultured at 37°C overnight in α-MEM containing 10% FBS (Sigma-Aldrich), 200 U/ml penicillin G, 200 μg/ml streptomycin, and 25 ng/ml M-CSF (R&D Systems). Non-adherent cells were combined in equal volumes with Ficoll density gradient solution (Sigma-Aldrich) and then were centrifuged at 2000 rpm for 30 min at room temperature. The cells in the middle layer were collected as BMMs. After washing with PBS, the cells were counted and inoculated in medium supplemented with 25 ng/ml M-CSF and 5 ng/ml RANKL (R&D Systems).

### Lentivirus transfection

The TCIRG1-RNAi lentivirus was provided by Shanghai Jikai Gene Chemical Technology Co. Ltd. CN. BMMs were transduced in transfer solution containing 5 μg/ml polybrene with a viral load of 20 times the number of cells. Ten hours after transfection, the virus was removed, and 48 h after transfection, the cells were induced to osteoclasts. Empty lentivirus was used as control.

### TRAP staining

After 96 h induction with RANKL, the cells were fixed in 4% formaldehyde for 20 min at room temperature, and TRAP staining was performed with acid phosphatase and a leukocyte kit (Sigma-Aldrich) according to the protocol. The number of TRAP-positive cells was counted under a light microscope at 20× magnification. The experiments were performed at least three times.

### Intracellular Ca^2+^ oscillation measurement

Cells were seeded into confocal microscopy dishes and induced to differentiate with RANKL. After 48 hours, the cells were incubated in 5 μM Fluo-3AM (Beyotime) and 0.05% Pluronic F-127 (Sigma-Aldrich) for 30 min at 37°C. The cells were washed three times with Hanks balanced salt solution and excited at 488 nm under confocal microscopy. The intracellular calcium oscillation was recorded for 3 min at 1 s intervals.

### Immunofluorescence staining

BMMs were seeded on coverslips and induced to differentiate for 4 days. Then, the cells were fixed with 4% formaldehyde for 20 min and permeabilized in 0.5% tritonX-100 solution for 5 min at room temperature. After blocking unspecific binding with 3% goat serum for 30 min at room temperature, the cells were incubated with LAMP2 rat monoclonal antibody (1:800, Santa Cruz, sc-20004) and V-ATPase V1B1&V1B2 rabbit anti mouse antibody (1:1000, Abcam, ab20839) overnight at 4°C. The cells were then washed and incubated with anti-rat secondary antibody conjugated to Alexa Fluor® 647 (1:1000, Abcam, ab150167) and anti-rabbit secondary antibody conjugated to Alexa Fluor® 568 (1:1000, Abcam, ab175471) for 1 h before observation.

### Immunohistochemistry of NFATc1

Cells were seeded on coverslips and induced to differentiate for 4 days. The cells were then fixed with 4% formaldehyde at room temperature for 20 min and permeabilized in 0.5% tritonX-100 solution for 5 min. To block nonspecific binding, coverslips were incubated in 3% goat serum at room temperature for 30 min and then with NFATc1 mouse monoclonal antibody (1:200, Santa Cruz, sc-7294) overnight at 4°C in PBS with 3% goat serum. After three washes in PBS, the cells were incubated with m-lgGκ BP-HRP antibody (1:1000, Santa Cruz, sc-516102) for 1 h at room temperature and then were counterstained with DAB (Cell signaling Technology).

### Western blot assays

Cells from 6-well culture plates were placed on ice and lysed with RIPA lysis buffer containing 1mM phenylmethylsulfonyl fluoride. Cell debris was removed by centrifugation at 12,000 rpm for 15 min at 4°C. Protein concentrations were measured using a BCA Protein Assay kit. Protein was denatured at 100°C for 10 min and then separated by 8% SDS-PAGE. The protein was transferred to polyvinylidene fluoride membranes, which were blocked with 5% BSA in Tris-buffered saline containing 0.1% Tween 20 (TBS-T) for 30 min and incubated with TCIRG1 rabbit polyclonal antibody (Abcam, ab139812), NFATc1 mouse monoclonal antibody (Santa Cruz, sc-7294), IP3R1 mouse monoclonal antibody (Santa Cruz, sc-271197) or IP3R2 mouse monoclonal antibody (Santa Cruz, sc-398434) at 4°C overnight. The membranes were washed three times with TBS-T for 10 min. After incubation with secondary antibodies for 1 h at room temperature, the membranes were washed three times with TBS-T for 5 min each, and images were detected and analyzed using a luminescent image analyzer (Tanon, Shanghai, CN).

Cytoplasmic protein and nuclear protein were harvested with the Nuclear and Cytoplasmic Protein Extraction Kit (Beyotime), according to the protocol. After Western blotting, nuclear protein was quantified by Lamin B1 (Abcam, ab133741).

### RT-PCR

Total RNA was extracted using TRIzol reagent, and 1 μg of RNA was reverse transcribed to cDNA using a PrimeScript^TM^ RT reagent kit (Takara) according to the instructions. RT-PCR was performed with SYBR Premix Ex Taq (Takara). Primers (Table 1) were designed to have optimal annealing temperatures of 63 degrees. All RT-PCR data were normalized to the expression of *Gapdh*. Three separate experiments were performed.

**Table 1:** Primer sequences.

Tcirg1	Forward 5'-ATTGCCAGCTTTCGGGAGAC-3'
	Reverse 5'-CGGATCTTCTGTCCGATCTGC-3'
Nfatc1	Forward 5'-GGGTCAGTGTGACCGAAGAT-3'
	Reverse 5'-GGAAGTCAGAAGTGGGTGGA-3'
Dc-stamp	Forward 5'-AAAACCCTTGGGCTGTTCTT-3'
	Reverse 5'-GGCTGCTTTGATCGTTTCTC-3'
Cathepsin K	Forward 5'-CTTCCAATACGTGCAGCAGA-3'
	Reverse 5'-CCGAGCCAAGAGAGCATATC-3'
Mmp9	Forward 5'-CGTCGTGATCCCCACTTACT-3'
	Reverse 5'-AACACACAGGGTTTGCCTTC-3'
Ip3r1	Forward 5'-AACTGTGGGACCTTCACCAG-3'
	Reverse 5'-AACTCTCGCCAGTTTCTGGA -3'
Ip3r2	Forward 5'-GTTACAGGATGTCGTGGCCT-3'
	Reverse 5'-ATTCGCCGTAATGTGCTACC-3'
Ip3r3	Forward 5'-CAATGAGCACCACGAGAAGA -3'
	Reverse 5'-AACTTGACAGGGGTCACCAG -3'
Gapdh	Forward 5'-GGTTGTCTCCTGCGACTTCA-3'
	Reverse 5'-TGGTCCAGGGTTTCTTACTCC-3'

### Statistical analysis

Data are expressed as the mean ± standard deviation (SD) from at least 3 different experiments. Student’s *t*-test was used for statistical analysis. * P < 0.05 and ** p < 0.01 were considered statistically significant.

## Results

### *Tcirg1* knockdown limits large-osteoclast genesis in BMMs

**Fig 1:**
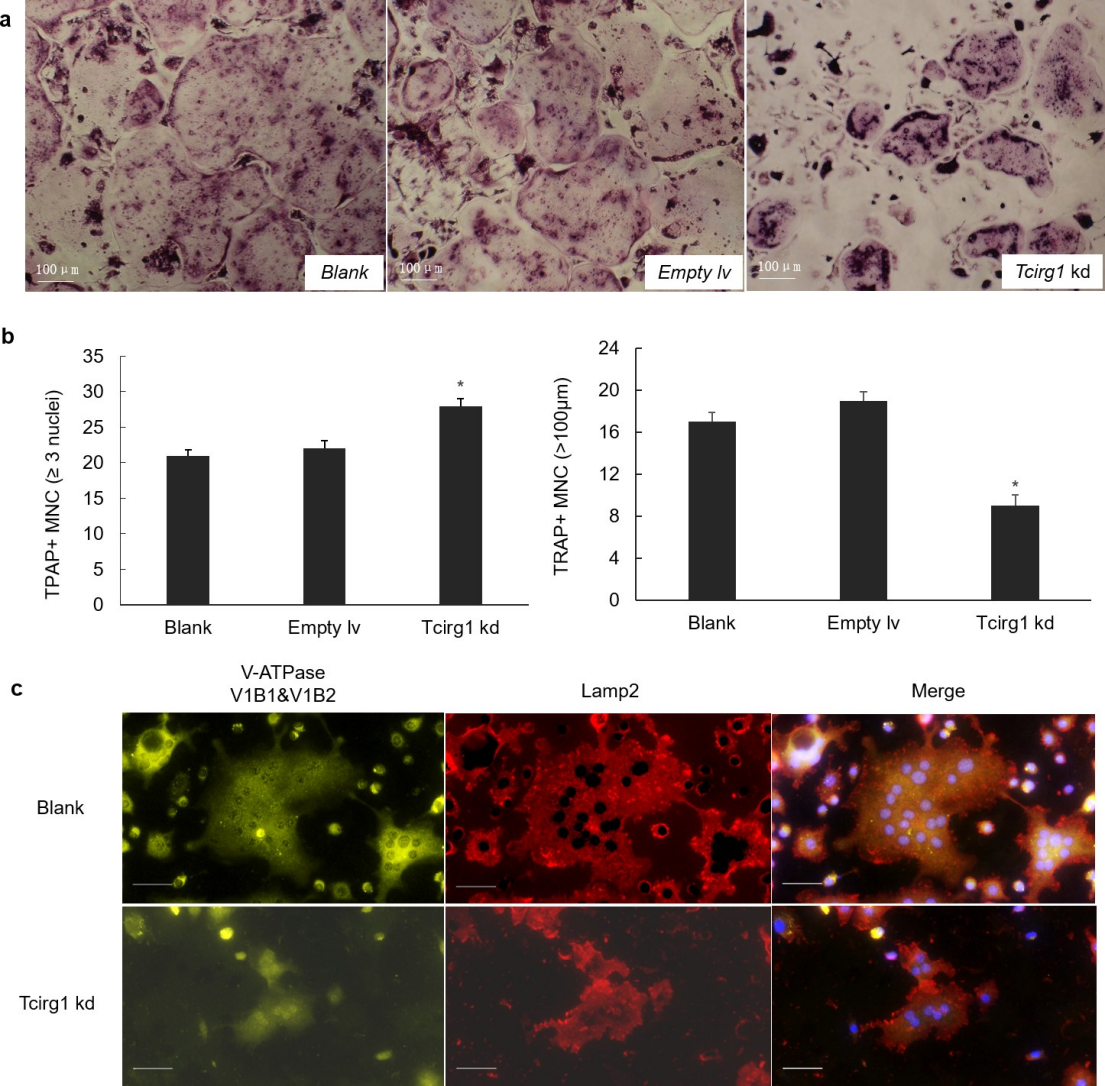
Knockdown of *Tcirg1* gene limits large-osteoclast genesis in BMMs. (a) BMMs that were treated with RANKL and M-CSF for 96 h were fixed and stained for TRAP. (b) TRAP-positive multinucleated cells (MNCs) with more than 3 nuclei (left panel) and TRAP-positive MNCs with a volume larger than 100 μm (right panel) were counted. The values are expressed as mean ± SD (n = 3) with untreated cells (blank) as control. (c) Immunofluorescence staining of LAMP2 and V-ATPase V1B1&V1B2. Results show that V-ATPase was localized in the lysozymes in the control and knockdown cells, but that there was less overall expression in the knockdown cells. Scale bars represent 20 μm.

To characterize the role of Tcirg1 in osteoclast formation, we transduced mouse BMMs with an empty lentiviral vector or a lentiviral vector expressing a *Tcirg1* RNAi. BMMs were treated with RANKL and M-CSF to induce osteoclast differentiation. After 96 h, they were stained for TRAP (Fig 1A). The number of TRAP-positive multinucleated cells (MNCs) with 3 or more nuclei were increased in the *Tcirg1* knockdown osteoclasts as compared to untreated cells (Blank) (P = 0.023), though the number of TRAP-positive MNCs with a volume larger than 100 μm was significantly decreased (P = 0.035) (Fig 1B). These findings are consistent with those of Li. et al [6]. To verify these results, the BMMs were induced to osteoclasts for 96 h and then were stained for LAMP2 and V-ATPase V1B1&V1B2. As shown in Fig 1C, *Tcirg1*-knockdown BMMs generated smaller osteoclasts. The staining for Lamp2 and V-ATPase V1B1&V1B2 was much weaker, but the V-ATPase was still localized to the lysosome. These results suggest that *Tcirg1* knockdown decreases Lamp2 and V-ATPase expression, leading to smaller osteoclasts.

### Expression and nuclear translocation of NFATc1 are decreased in *Tcirg1*-knockdown osteoclasts

**Fig 2:**
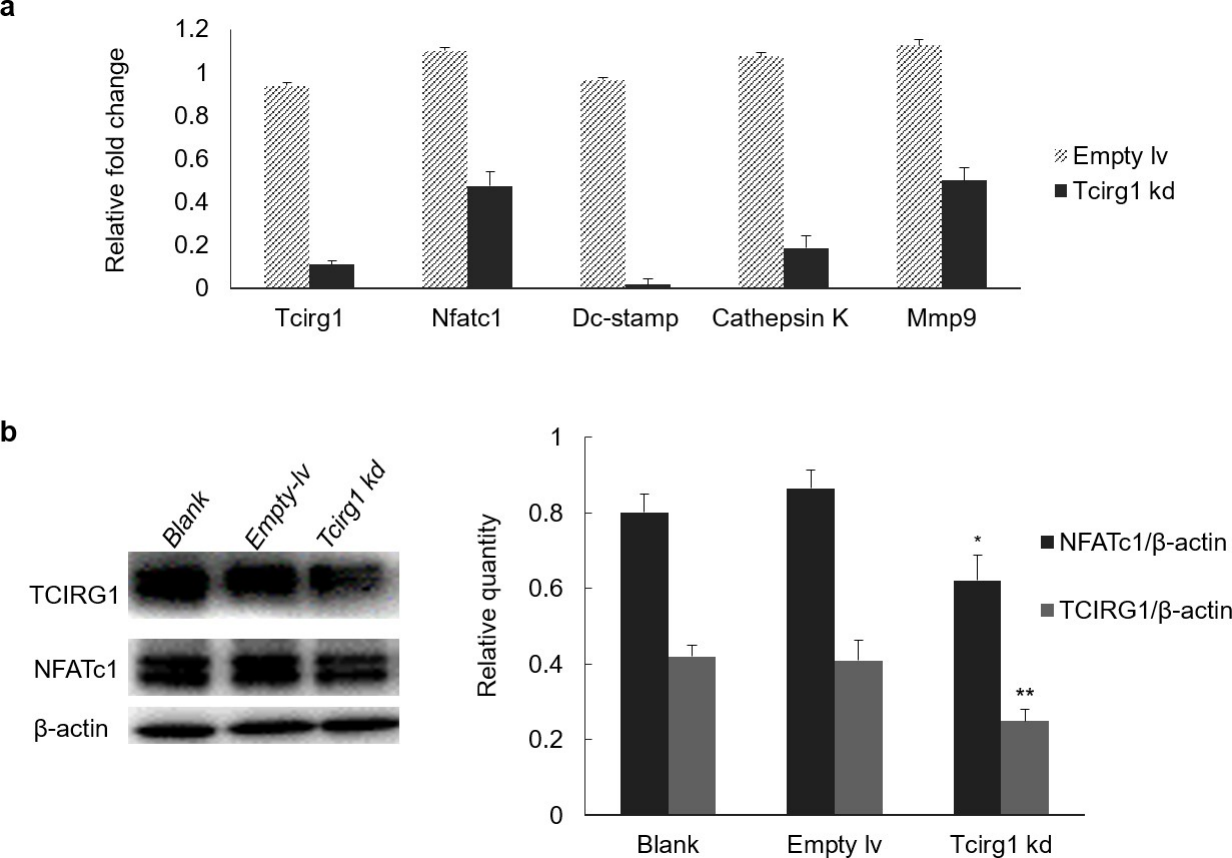
Expression of NFATc1 is decreased in *Tcirg1*-knockdown osteoclasts. (a) Cells treated with RANKL and M-CSF for 48h were collected, and RT-PCR was performed to determine the mRNA expression of *Nfatc1*, *Dc-stamp*, *Cathepsin K*, and *Mmp9*. (b and c) Expression of TCIRG1 and NFATc1 proteins were determined by western blotting using cells treated with RANKL for 48 h. Data are normalized to β-actin expression, and untreated cells (blank) were compared as control. The values are expressed as mean ± SD, n = 3.

To assess whether *Tcirg1* knockdown also affects the expression levels of osteoclast-specific genes, we collected osteoclasts after treatment with RANKL for 48 h. RT-PCR results showed that mRNA expression of genes related to osteoclast differentiation, such as *Tcirg1*, *Nfatc1*, *Dc-stamp*, *Cathepsin K* and *Mmp9*, were down-regulated (Fig 2A). Western blotting results, which are consistent with the mRNA results, verified a corresponding decrease in the expression of NFATc1 and TCIRG1 (Fig 2B and 2C, NFATc1 P = 0.037, TCIRG1 P = 0.009). To determine whether the effect of lentivirus was persistent, the mRNA expression of *Tcirg*1, *Nfatc1*, *Dc-stamp*, *Cathepsin K* and *Mmp9* genes was evaluated by RT-PCR. The results verified persistent inhibition of these genes (S1 Fig).

**Fig 3:**
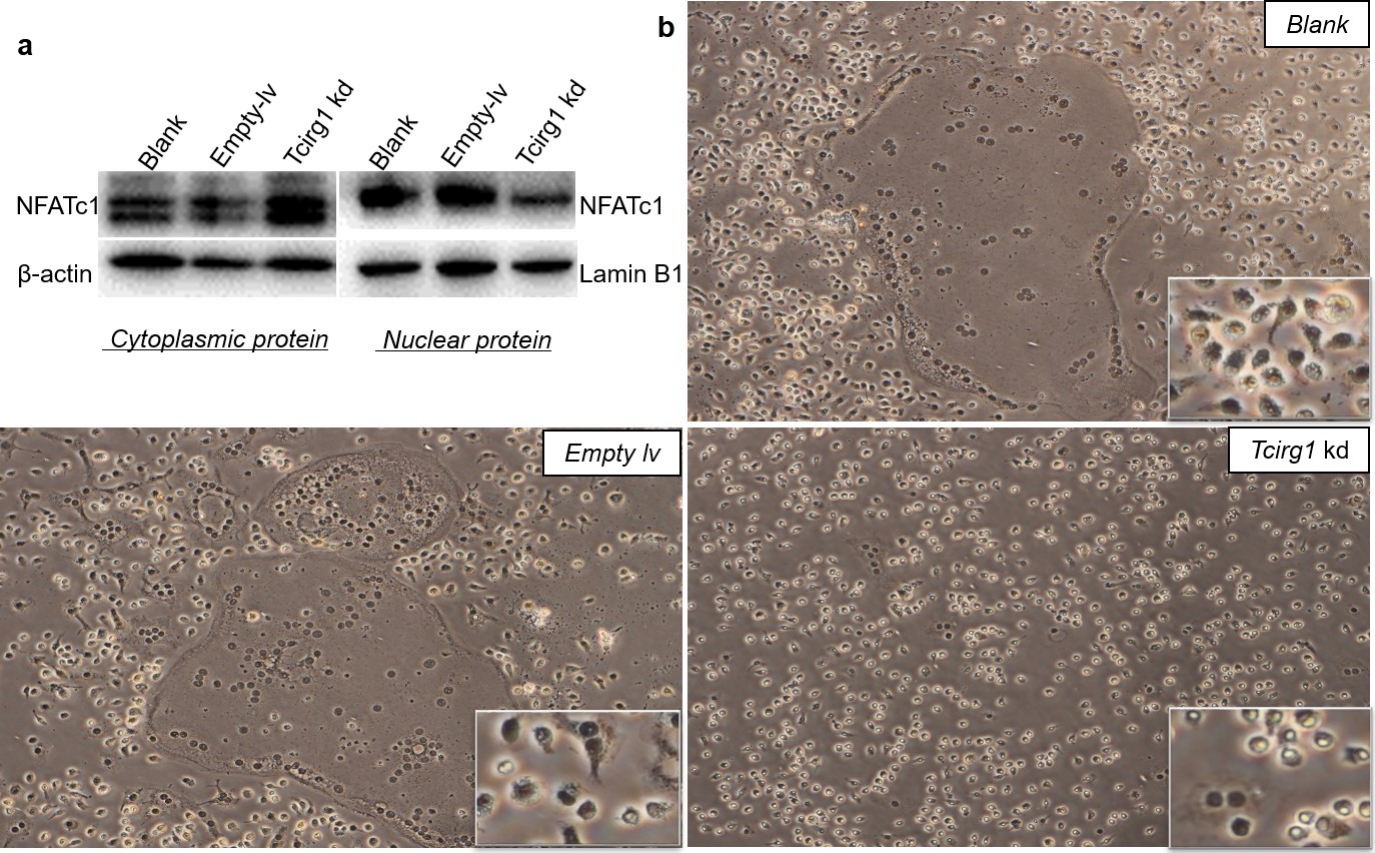
Nuclear translocation of NFATc1 is decreased in *Tcirg1*-knockdown osteoclasts. (a) Nuclear and cytoplasmic levels of the NFATc1 protein were measured by western blotting using cells treated with RANKL for 48 h. (b) Immunohistochemistry of NFATc1. The *Tcirg1-* knockdown osteoclasts were smaller and had more negative nuclei.

As a key factor in osteoclastogenesis, NFATc1is translocated to the nucleus, where it exerts transactivation functions [12]. Therefore, we collected cells after treating them with RANKL for 48 h and extracted cytoplasmic and nuclear proteins separately for western blotting. As shown in Fig 3A, the nuclear translocation of NFATc1 was decreased in *Tcirg1-* knockdown cells. NFATc1 nuclear translocation was further analyzed by immunohistochemistry, which verified that *Tcirg1-* knockdown osteoclasts were smaller and had more NFATc1-negative nuclear (Fig 3B). Therefore, these results suggest that *Tcirg1* -knockdown reduces NFATc1 expression and translocation.

### Intracellular Ca^2+^ signaling and IP3R2 expression are decreased in *Tcirg1*-knockdown osteoclasts

**Fig 4:**
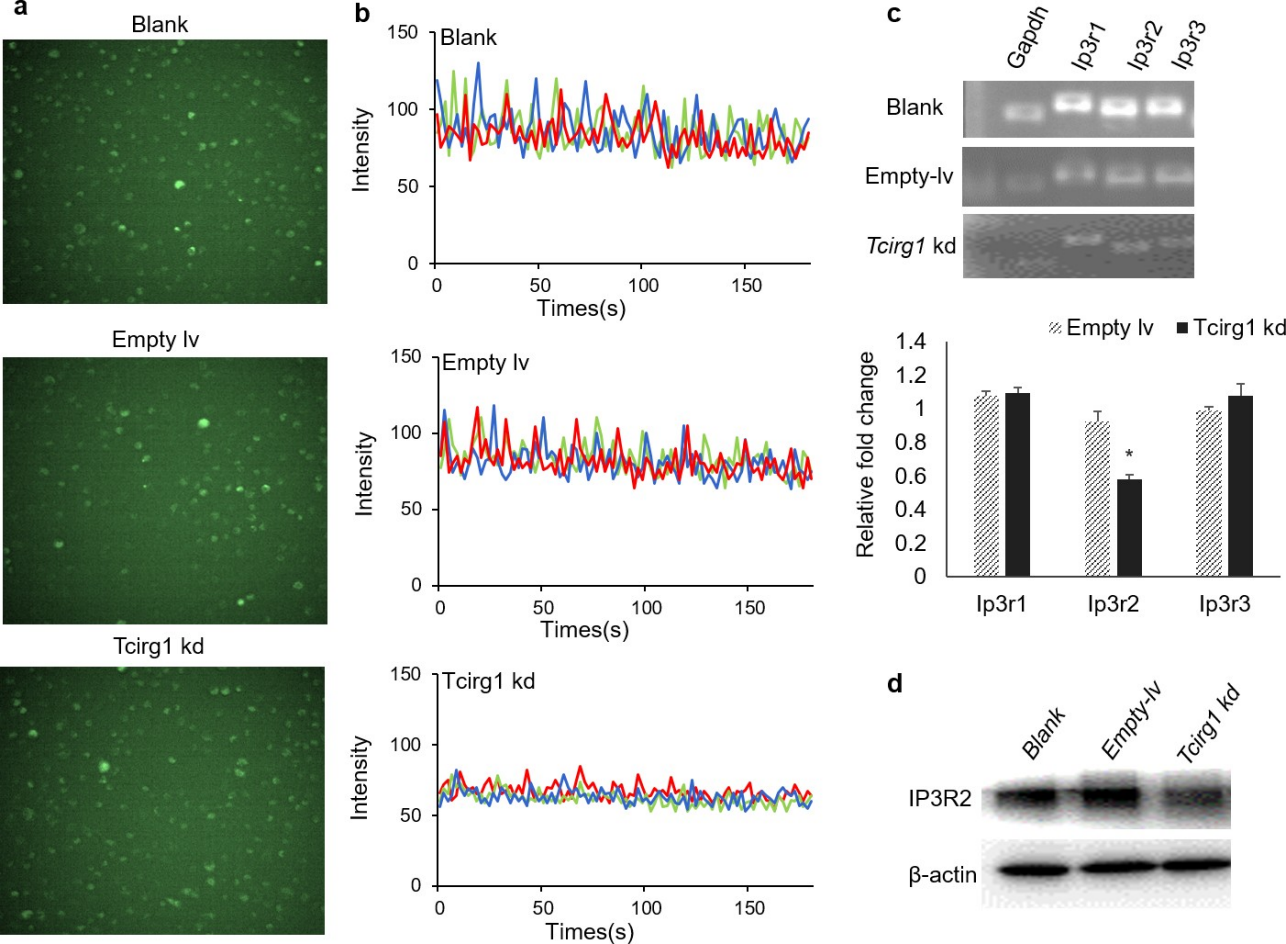
Intracellular Ca^2+^ signaling and IP3R2 expression are decreased in *Tcirg1*-knockdown osteoclasts. Fluo-4 fluorescent images of BMMs in different groups induced to differentiate for 48 h (a). Intracellular Ca^2+^ oscillations were measured by confocal microscopy at 1 s intervals for 180 s. Each color indicates a randomly chosen cell in the same field (b). RT-PCR and Western blot assays were performed to determine the mRNA expression (c) and protein levels (d) of IP3Rs.

The nuclear translocation of NFATc1 is regulated by Ca^2+^ oscillation [13]. Therefore, we measured the intracellular Ca^2+^ oscillation in different groups of cells by confocal microscopy at 1 s intervals for 180 s after the cells were induced to differentiate for 48 h (Fig 4A and 4B). The results suggest that knockdown of *Tcirg1* inhibits both the average amplitude and the frequency of Ca^2+^ oscillation.

IP3Rs, which mediate the release of intracellular calcium ions in response to extracellular signals, include three subtypes: IP3R1, IP3R2, and IP3R3 [14]. To investigate whether knockdown of *Tcirg1* regulates intracellular Ca^2+^ signaling by affecting the expression of IP3Rs, we performed RT-PCR and western blot assays after treatment with RANKL for 48 h. Expression of all three receptors of IP3Rs was detectable in RANKL-induced BMMs, whereas knockdown of the *Tcirg1* gene caused decreased expression of IP3R2, but not IP3R1 or IP3R3 (Fig 4C and 4D, n = 3, P = 0.021). These results suggest that the inhibition of NFATc1 translocation by *Tcirg1* knockdown may be mediated by decreased Ca^2+^ signaling through the IP3R2 receptor.

## Discussion

*TCIRG1* encodes V-ATPase a3 subtype, and its mutation is responsible for human recessive osteopetrosis [15, 16]. V-ATPase is widely distributed in various types of cells. In osteoclasts, V-ATPase is mainly of the d2/a3 subtype, which is crucial to the fusion of osteoclast precursors and the function of mature osteoclasts [3]. To characterize the molecular function of TCIRG1, we knocked down the *Tcirg1* gene in BMMs. Our results demonstrate that in RANKL-induced osteoclasts, the number of large osteoclasts (>100 μm) decreased, while the number of TRAP-positive cells increased. These findings may be related to the blockade of cell fusion in *Tcirg1*-knockdown mice.

The transcription factor NFATc1 is known to be potently induced by RANKL [13]. Furthermore, RANKL can induce sustained Ca^2+^ oscillation in BMMs and activate NFATc1, which translocates to the nucleus upon activation [17]. NFATc1 directly binds to the promoter region of osteoclast-related genes, such as *Mmp9*, *Cathepsin K*, and *Trap*, to induce osteoclastogenesis [12, 17, 18]. In this study, we found that the expression and nuclear translocation of NFATc1 were decreased in *Tcirg1*-knockdown osteoclasts. Furthermore, the expression of *Dc-stamp* and *cathepsin K* were reduced. DC-STAMP is important to pro-osteoclast fusion, and its gene expression is promoted by NFATc1 [19]. The down-regulation of Dc-stamp may contribute to the production of smaller osteoclasts in *Tcirg1*-knockdown BMMs. Additionally, NFATc1 has been shown to activate Cathepsin K expression through its interaction with PU.1 in nuclei [20]. Therefore, NFATc1 induction of DC-STAMP and Cathepsin K may contribute to the inhibited osteogenesis in *Tcirg1*-knockdown BMMs.

IP3Rs are transmembrane proteins that localize to the ER and function as calcium ion-releasing channels [14]. While all 3 subtypes mediate regulate Ca^2+^ oscillation, IP3R2 is required for persistent Ca^2+^ oscillation, and IP3R3 mediates only single-phase Ca^2+^ transients [21]. Persistent Ca^2+^ oscillation is necessary for nuclear translocation of NFATc1 during the early stage of osteoclastogenesis [22]. In this study, we found that the expression of IP3R2 was decreased in *Tcirg1*-knockdown osteoclasts and that the level of intracellular Ca^2+^ oscillation was also decreased, suggesting that the reduced NFATc1 nuclear translocation levels may be explained by reduced intracellular Ca^2+^.

The increase of [Ca^2+^]_i_ primarily depends on the release of intracellular calcium stores, followed by the reabsorption of extracellular Ca^2+^, namely Store-operated calcium entry (SOCE) [23]. Studies have shown that the loss of IP3Rs leads to reduced intracellular Ca^2+^ levels in BMMs, suggesting that IP3Rs are necessary for the release and inflow of Ca^2+^ [11]. Transient receptor potential (TRPs) cation channels, including TRPC, TRPV, TRPM, TRPA, TRPP and TPRML subfamilies, are considered to be permeable calcium channels. Depletion of intracellular calcium stores induces or mediates the activity of TRP channels, and the increase of [Ca^2+^]_i_ inhibits their function [24]. TRPV4 has been shown to be related to osteoclast differentiation: TRPV4 depletion leads to osteoclast maturation defects with no apparent effect on intracellular calcium oscillation, suggesting that TRPV4 does not regulate intracellular calcium oscillation during osteoclast differentiation [25]. Thus, whether [Ca^2+^]_i_ reduction in *Tcirg1* knockdown cells (S2 Fig) is related to TRPs needs further investigation.

In our study, knockdown of the *Tcirg1* gene downregulated the expression of V-ATPase V1B1+ V1B2 and LAMP2 in RANKL-induced osteoclasts. However, there are few studies on TCIRG1 and lysosome generation to support these findings. In recent years, the translation factor EB (TFEB) has been identified as a transcription factor regulating lysosomes generation [26]. In *Tfeb*^*-/-*^ mice, osteoclasts are normally generated but are functionally deficient, with fewer and smaller lysosomes [27]. *Tfeb* over-expression increases the number of autophagosomes, enhances the fusion of lysosomes and autophagosomes, and degrades proteins that have high stability [28]. TFEB activity is regulated by various factors, and usually TFEB is phosphorylated in the cytoplasm [29, 30]. TFEB regulates its own expression and activity through a feedback loop during nutrient deficiency, which is rapid and stable [31]. The mechanistic target of rapamycin (mTOR) kinase has been shown to phosphorylate TFEB and to determine its localization in the cytoplasm [29, 30, 32]. Other studies have found that the effect of calcineurin on TFEB is strong, and that overexpression or continuous activation of calcineurin can cause persistent TFEB activation [33]. Inhibition of calcineurin activity suppresses TFEB activity, even under starvation conditions. Thus, upon inhibition of calcineurin and mTOR activity, TFEB remains in the cytoplasm, demonstrating that calcineurin has a stronger regulatory effect on TFEB than mTOR [33]. All of these considerations suggest the possibility that TFEB regulates the generation and function of lysosomes downstream of Tcirg1. Interestingly, TFEB has been found to regulate ATP6V0D1, V0D2 and V1C1 proteins in both HELA cells and mouse fibroblasts [26, 34], and reduced expression of Atp6v1c1 and Tcirg1 was also observed in *Tfeb*^*-/-*^ osteoclasts [27]. Whether knockdown of the *Tcirg1* gene adversely affects TFEB needs to be further explored.

## Conclusion

The current study shows that knockdown of the *Tcirg1* gene not only reduces the expression of NFATc1, but also inhibits calcium oscillation by reducing the expression of IP3R2, thus limiting the nuclear translocation of NFATc1 and leading to reduced expression of osteoclast-related genes. These findings provide a mechanism to explain the function of TCIRG1. Furthermore, on the basis of these findings, modulation of NFATc1 and IP3R2 expression and the use of calcium channel agonists may be considered for osteopetrosis therapy.

## Supporting information

S1 Fig Expression of osteoclast-related genes after cells were induced to osteoclasts for 96 h. RT-PCR was performed to determine the mRNA expression of *Tcirg1*, *Nfatc1*, *Dc-stamp*, *Cathepsin K*, and *Mmp9* after BMMs were induced to osteoclasts with RANKL for 96 h.
(PDF)

S2 Fig Intracellular Ca^2+^ levels. Cells were induced to differentiate for 48 h and then incubated with Fluo-3AM for 30 min. After three washes with PBS, cells were measured with a blue laser at 488 nm on a flow cytometer.
(PDF)

S1 Raw images (PDF)
