# Supplementary material for: Knockdown of Tcirg1 inhibits large-osteoclast generation by down-regulating NFATc1 and IP3R2 expression
Source: PLoS One. 2020 Aug 13;15(8):e0237354. doi: 10.1371/journal.pone.0237354 (PMC7425954; doi:10.1371/journal.pone.0237354)

S1 Fig. Expression of osteoclast-related genes after cells were induced to osteoclasts for 96 h.

RT-PCR was performed to determine the mRNA expression of *Tcirg1*, *Nfatc1*, *Dc-stamp, Cathepsin K,* and *Mmp9* when BMMs were induced to osteoclasts with RANKL for 96 h.

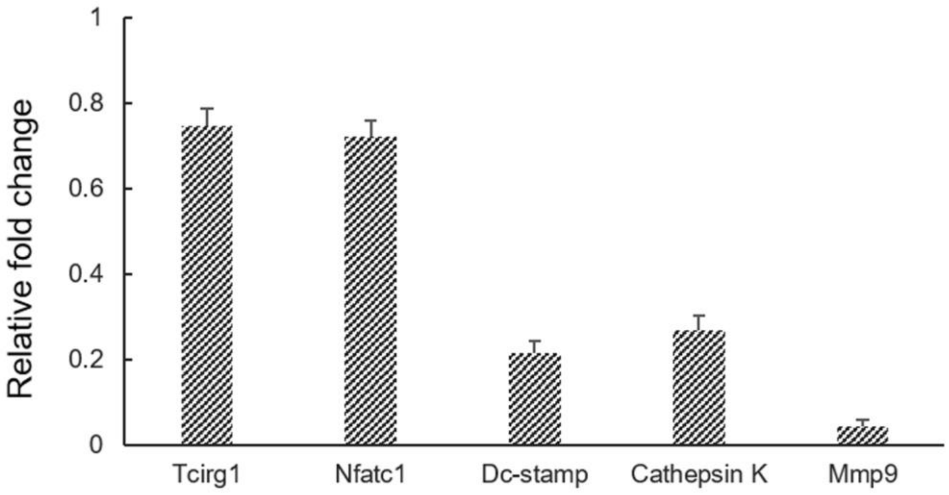

Supplement: S1 Fig — RT-PCR was performed to determine the mRNA expression of Tcirg1, Nfatc1, Dc-stamp, Cathepsin K, and Mmp9 after BMMs were induced to osteoclasts with RANKL for 96 h. (PDF) [file pone.0237354.s001.pdf]
