# Supplementary material for: Knockdown of Tcirg1 inhibits large-osteoclast generation by down-regulating NFATc1 and IP3R2 expression
Source: PLoS One. 2020 Aug 13;15(8):e0237354. doi: 10.1371/journal.pone.0237354 (PMC7425954; doi:10.1371/journal.pone.0237354)

**S2 Fig. Intracellular $Ca^{2+}$ levels.** Cells were induced to differentiate for 48 h and then incubated with Fluo-3AM for 30 min. After three washes with PBS, cells were measured by a blue laser at 488 nm on a flow cytometer.

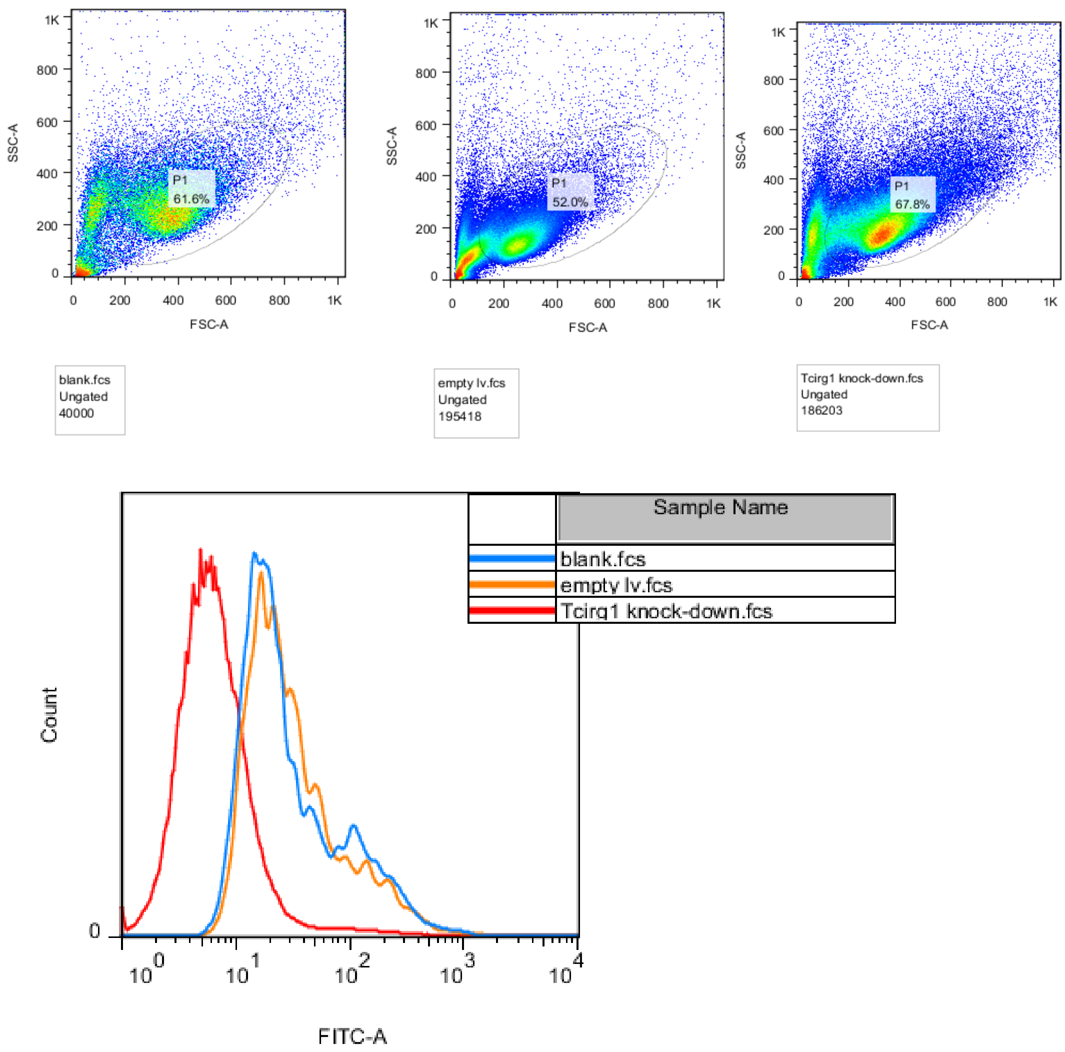

Supplement: S2 Fig — Cells were induced to differentiate for 48 h and then incubated with Fluo-3AM for 30 min. After three washes with PBS, cells were measured with a blue laser at 488 nm on a flow cytometer. (PDF) [file pone.0237354.s002.pdf]
