# Supplementary material for: Knockdown of Tcirg1 inhibits large-osteoclast generation by down-regulating NFATc1 and IP3R2 expression
Source: PLoS One. 2020 Aug 13;15(8):e0237354. doi: 10.1371/journal.pone.0237354 (PMC7425954; doi:10.1371/journal.pone.0237354)

Total Protein

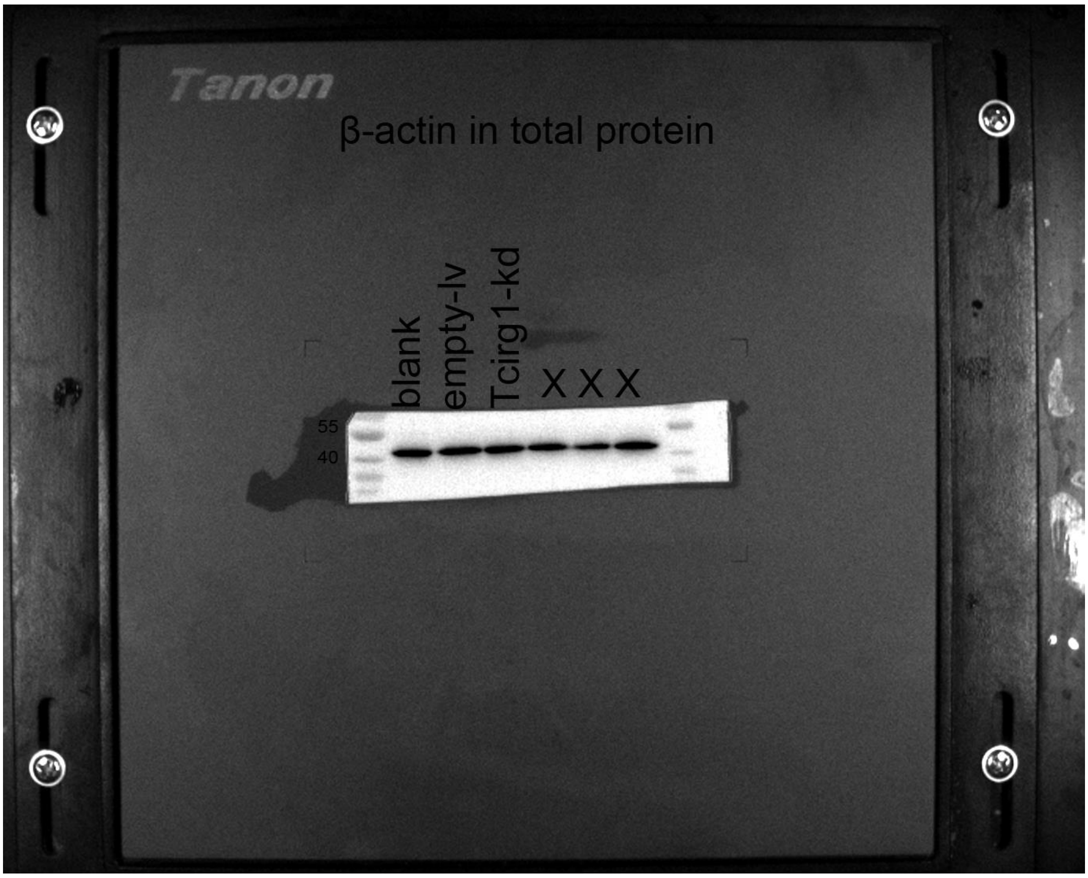

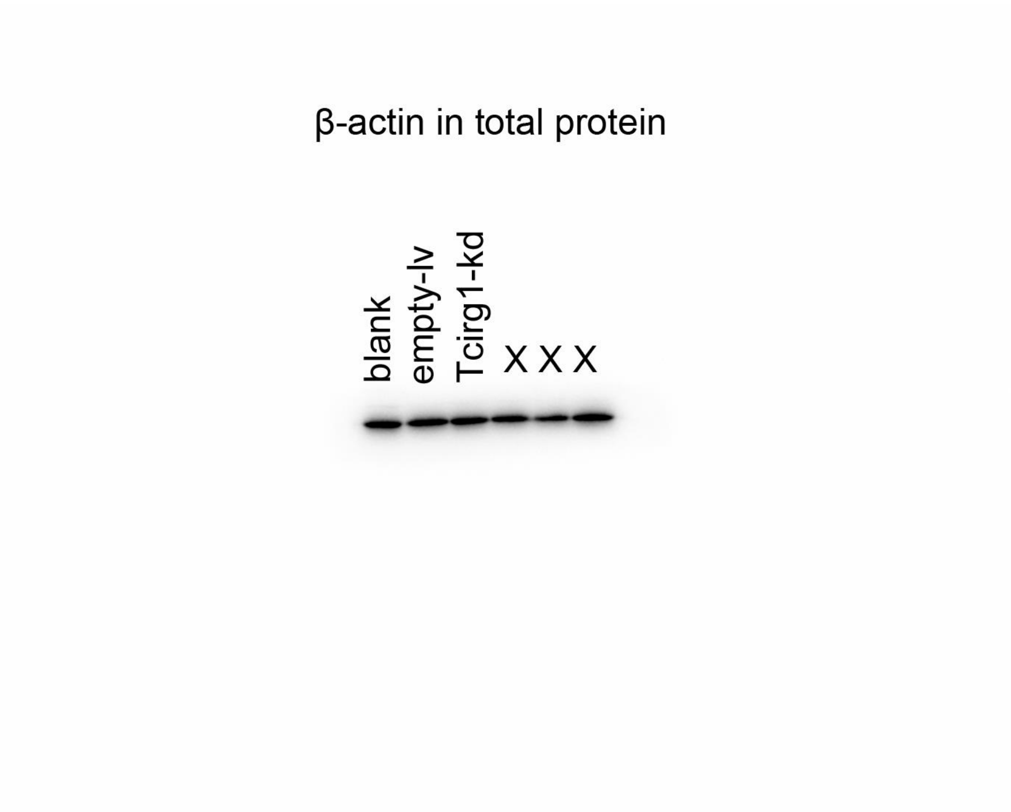

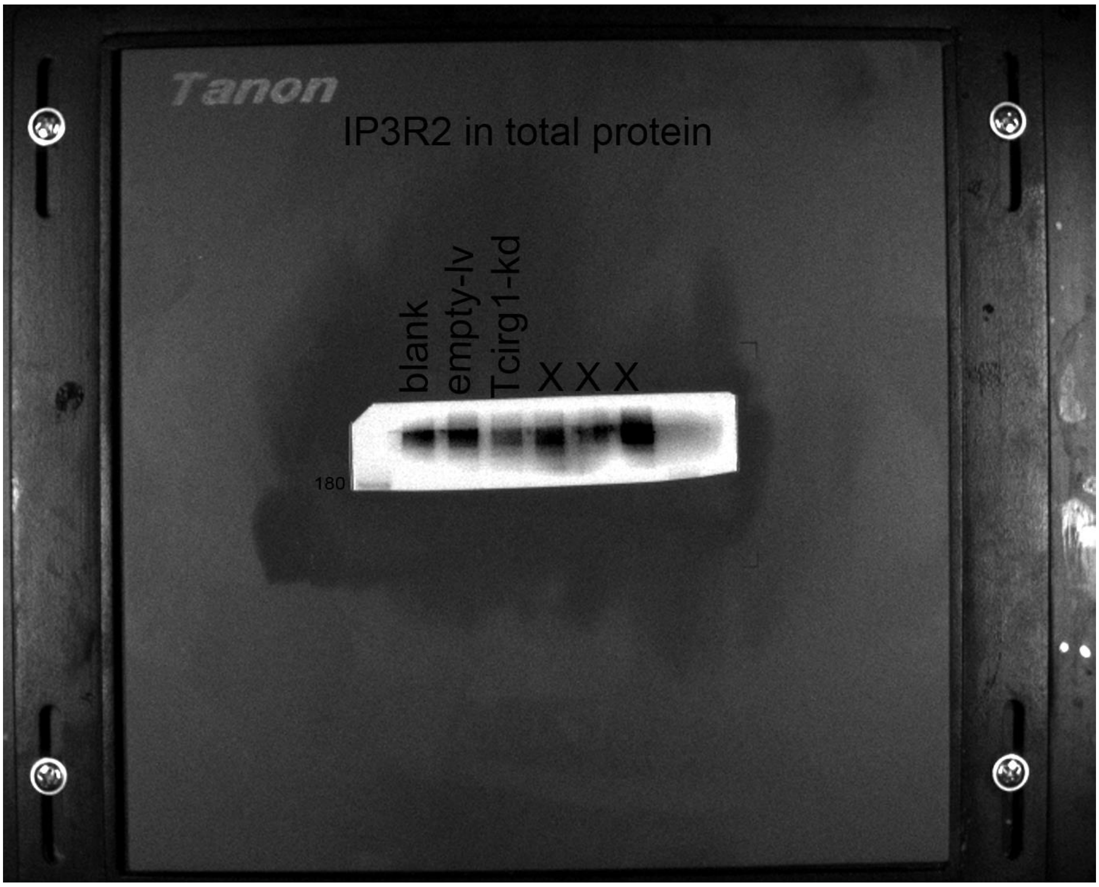

Tanon
IP3R2 in total protein
blank
empty-lv
Tcirg1-kd
X X X
180

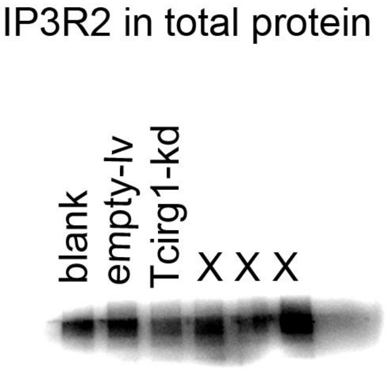

IP3R2 in total protein
blank
empty-lv
Tcirg1-kd
X X X

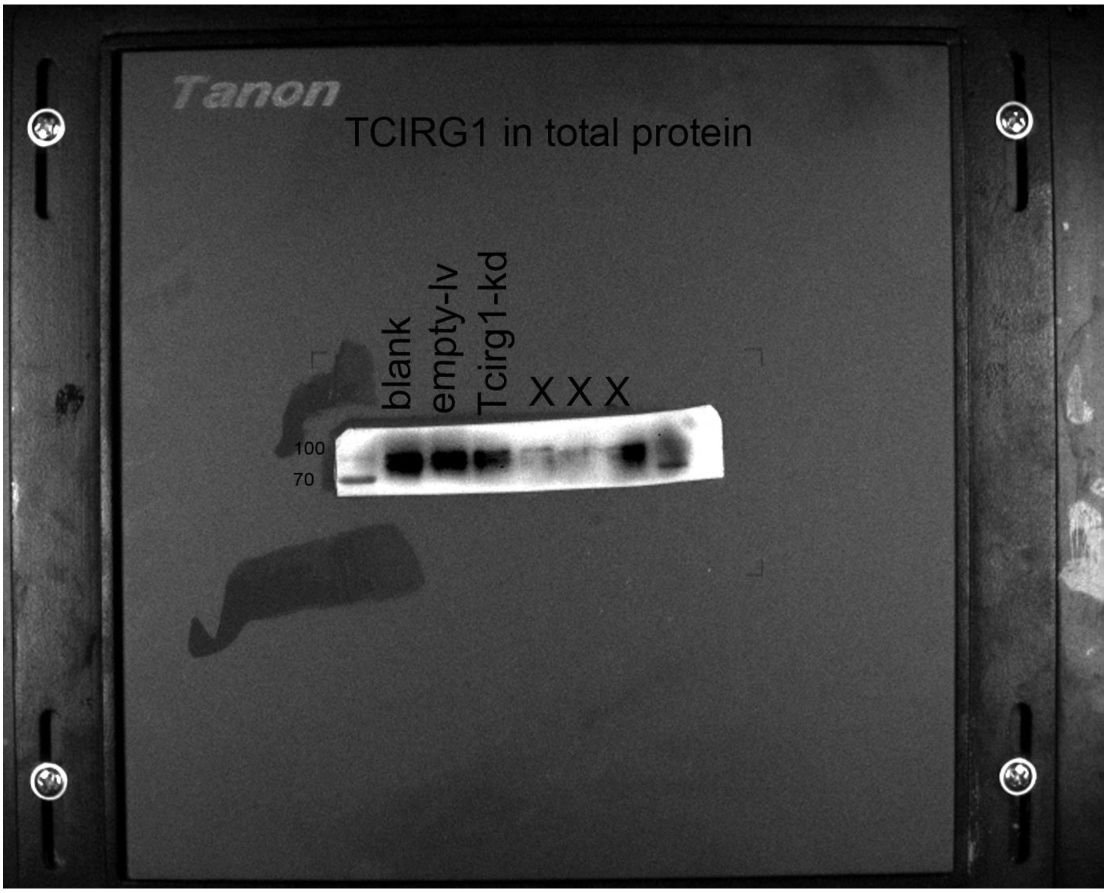
Tanon
TCIRG1 in total protein
blank
empty-lv
Tcirg1-kd
X X X
100
70

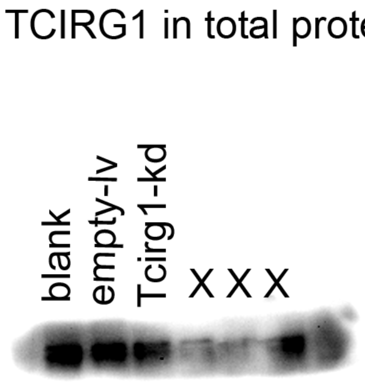
TCIRG1 in total protein
blank
empty-lv
Tcirg1-kd
X X X

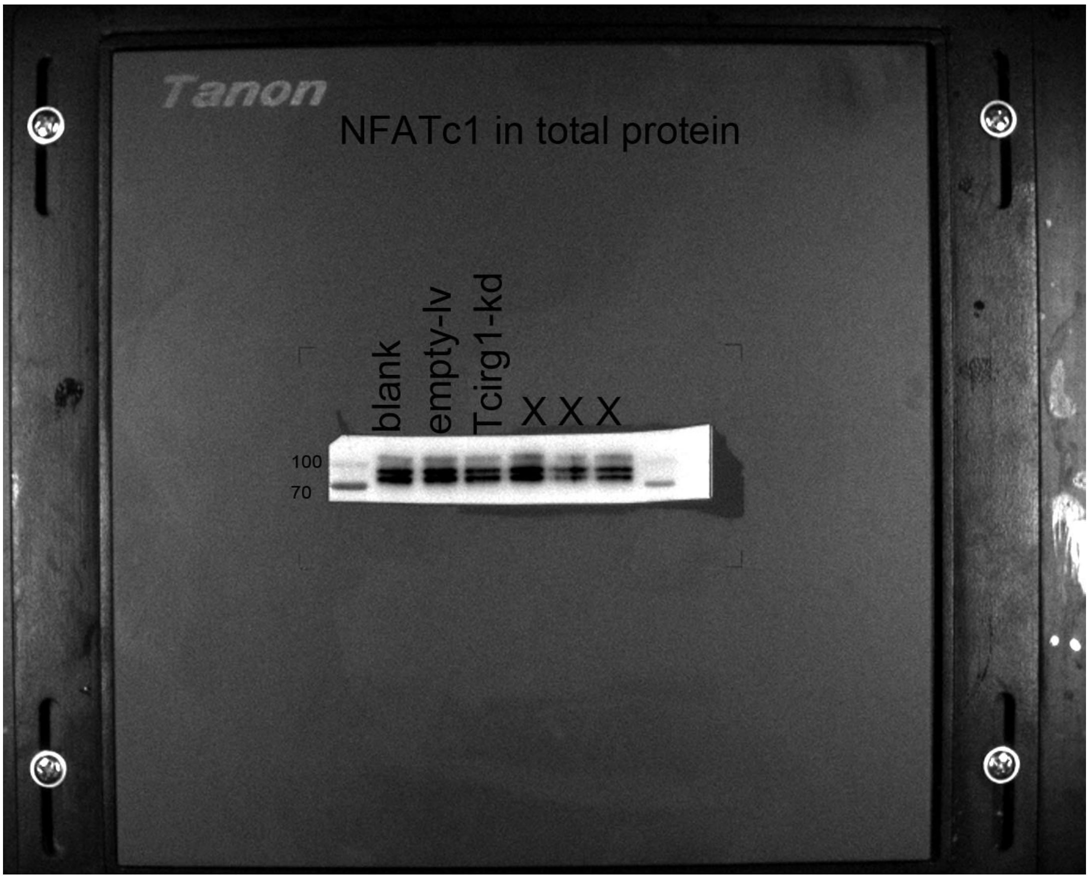

NFATc1 in total protein

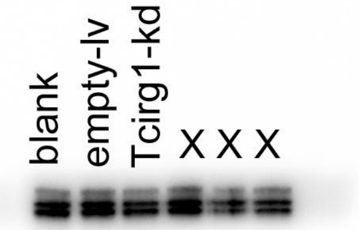

Cytoplasmic protein

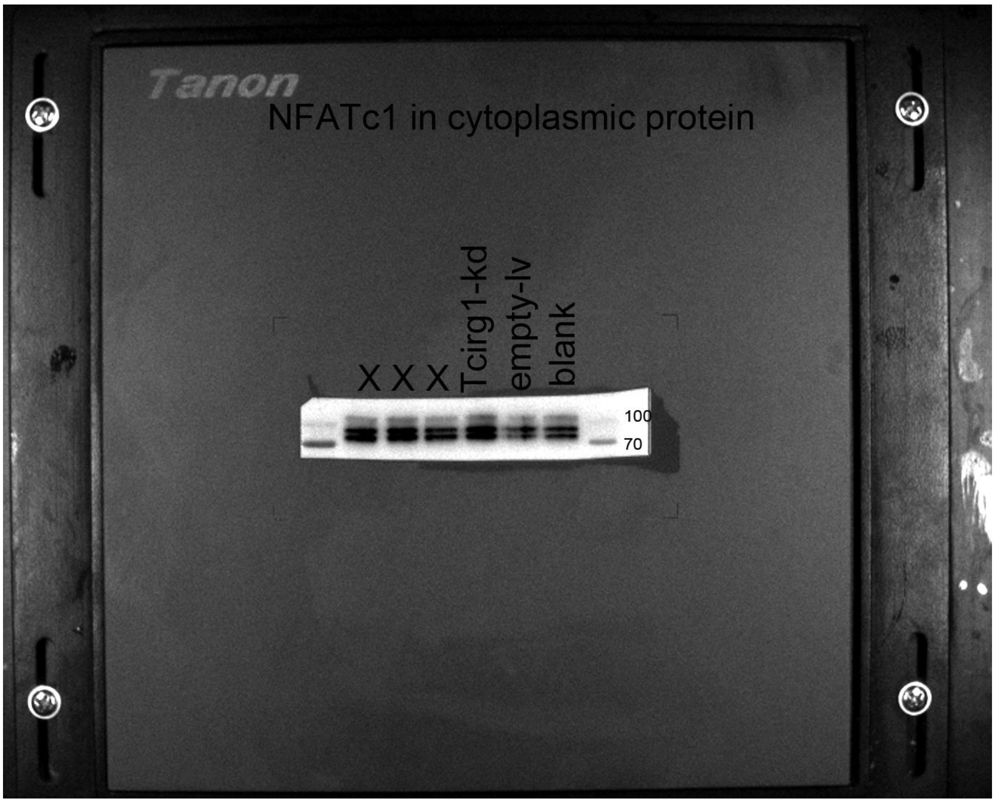

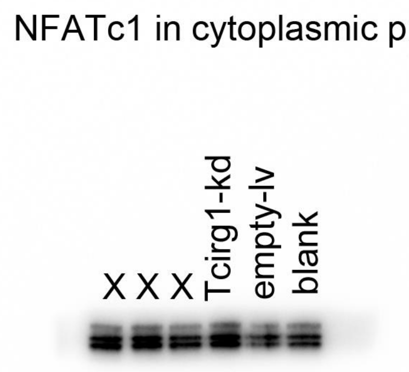

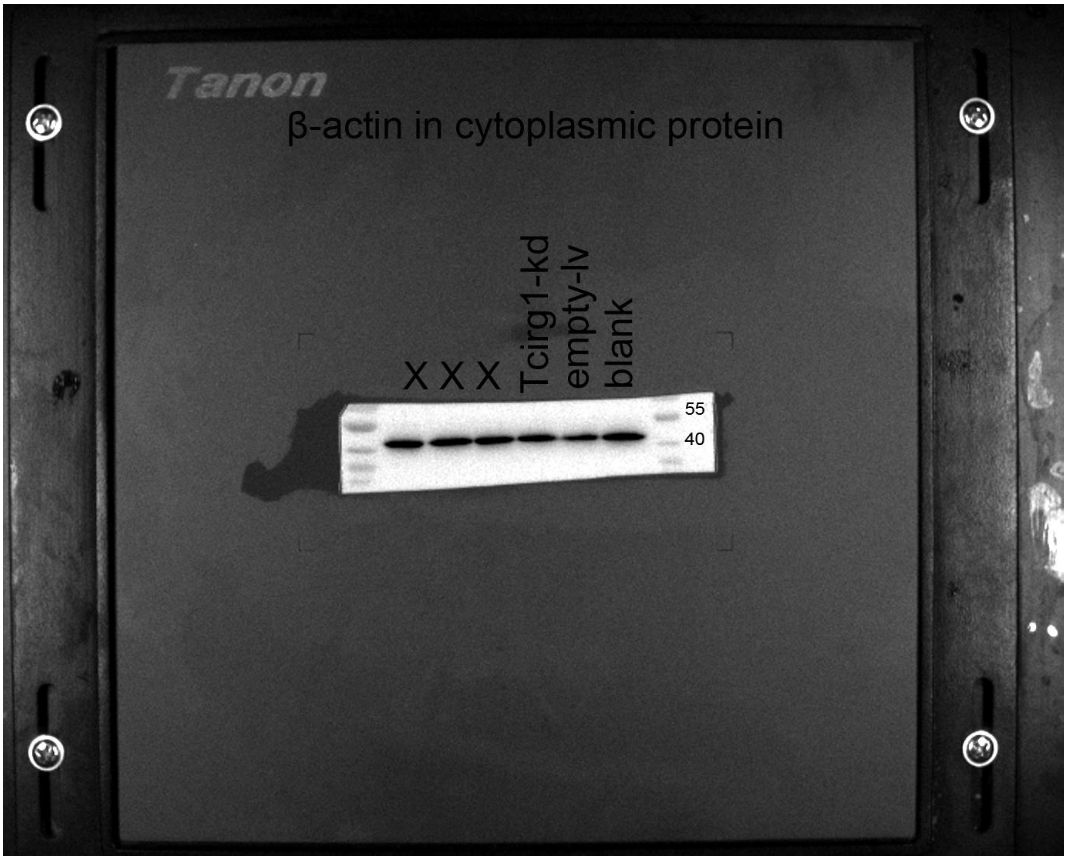
Tanon
β-actin in cytoplasmic protein
X X X
Tcirg1-kd
empty-lv
blank
55
40

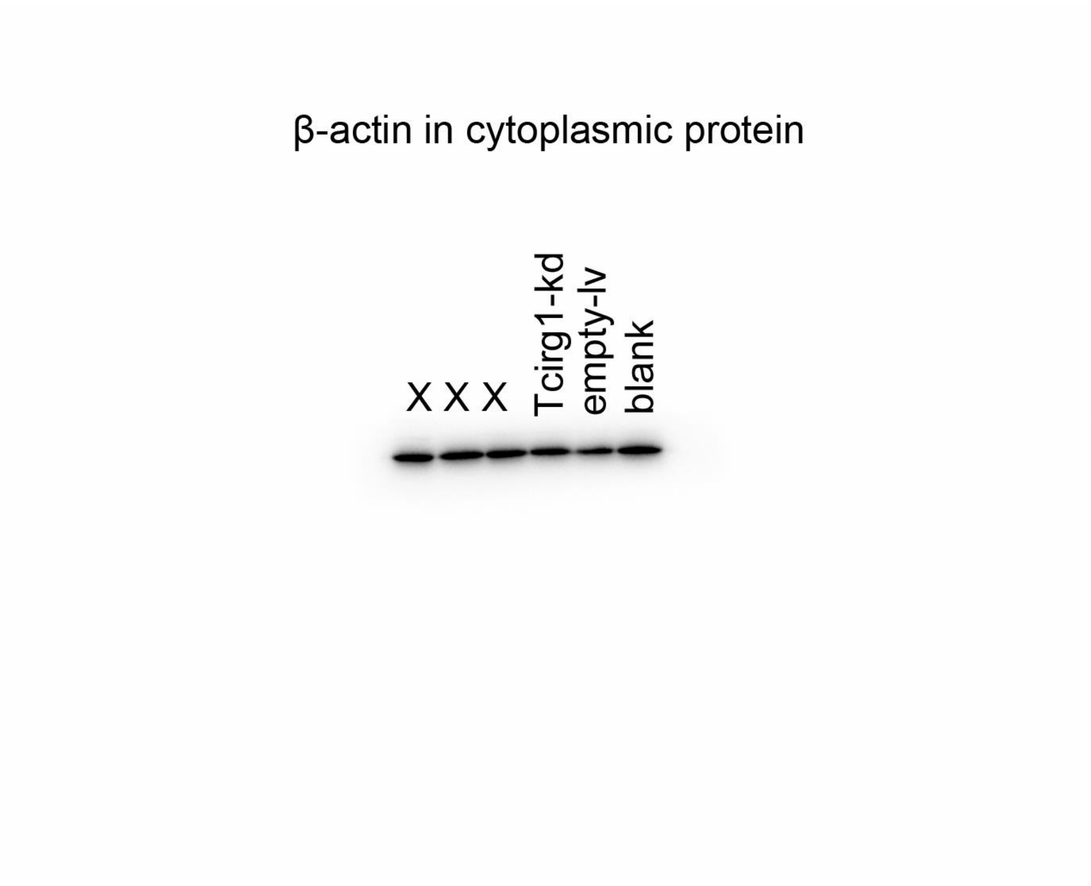
β-actin in cytoplasmic protein
X X X
Tcirg1-kd
empty-lv
blank

Nuclear protein

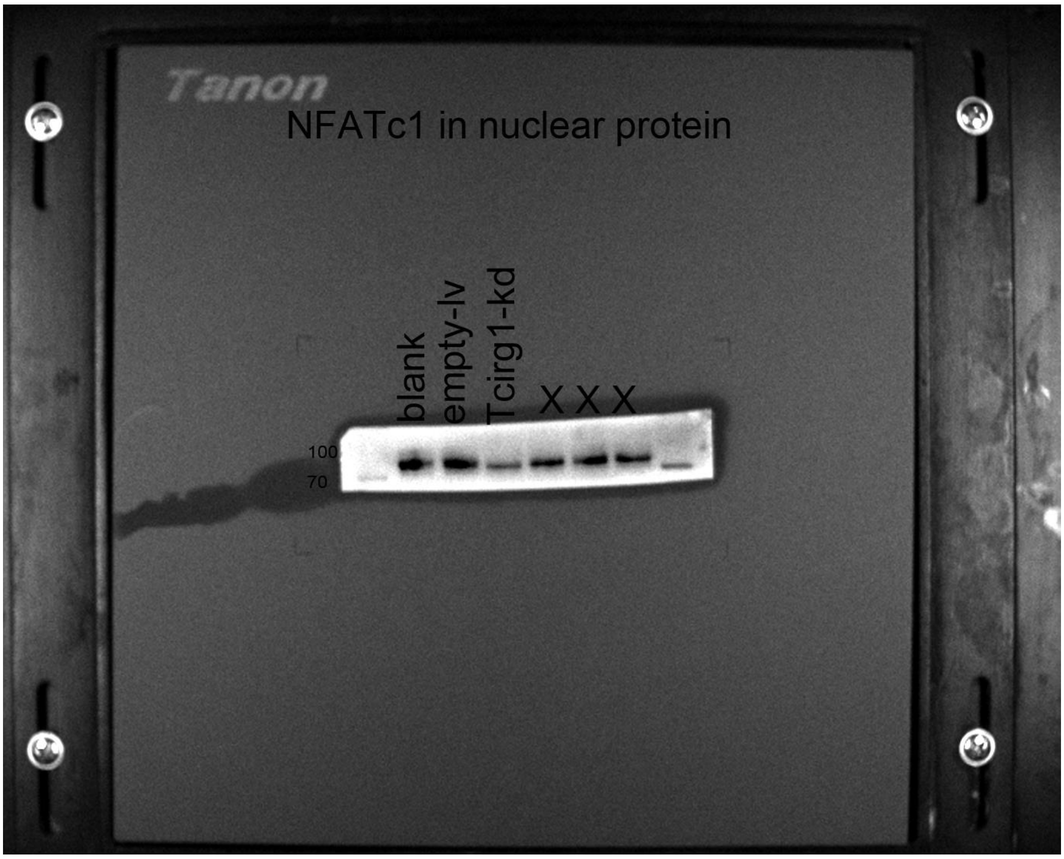

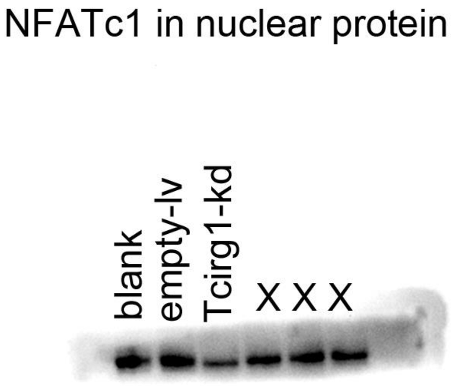

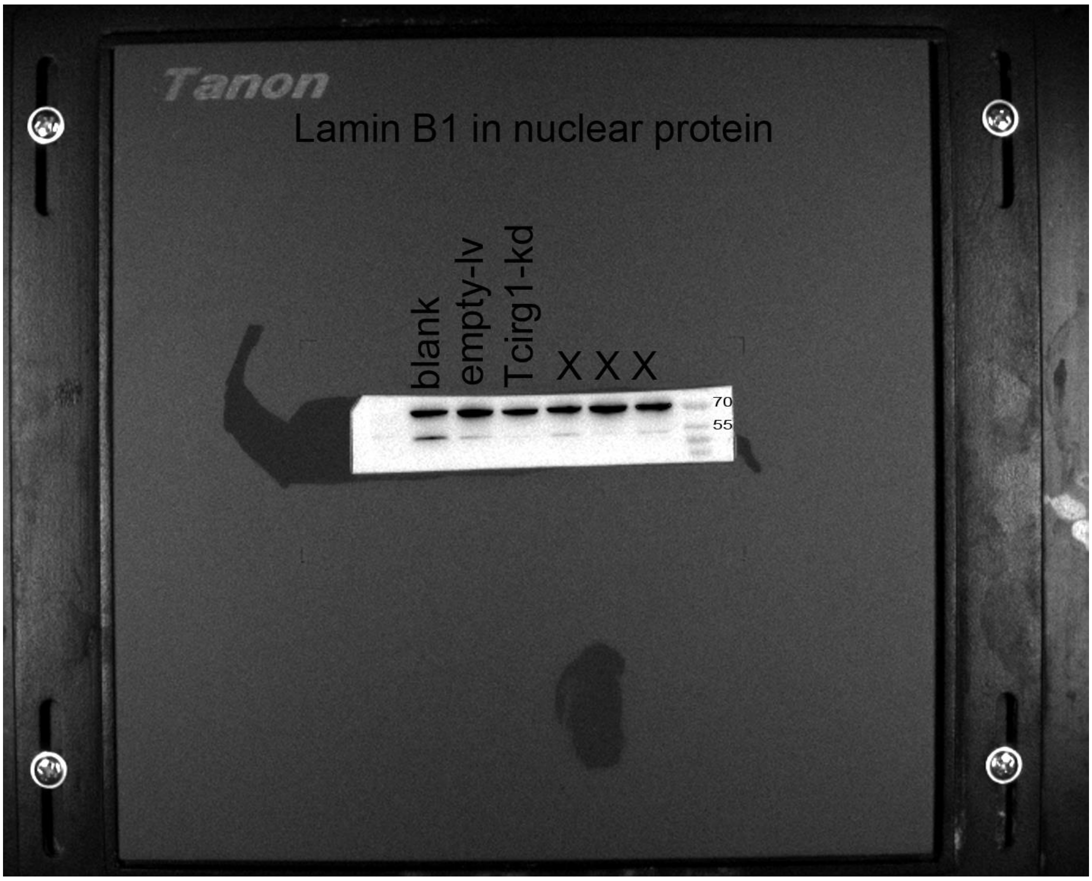

Lamin B1 in nuclear protein

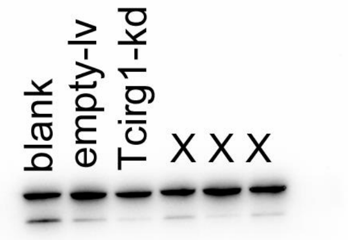

Agarose gel electrophoresis images

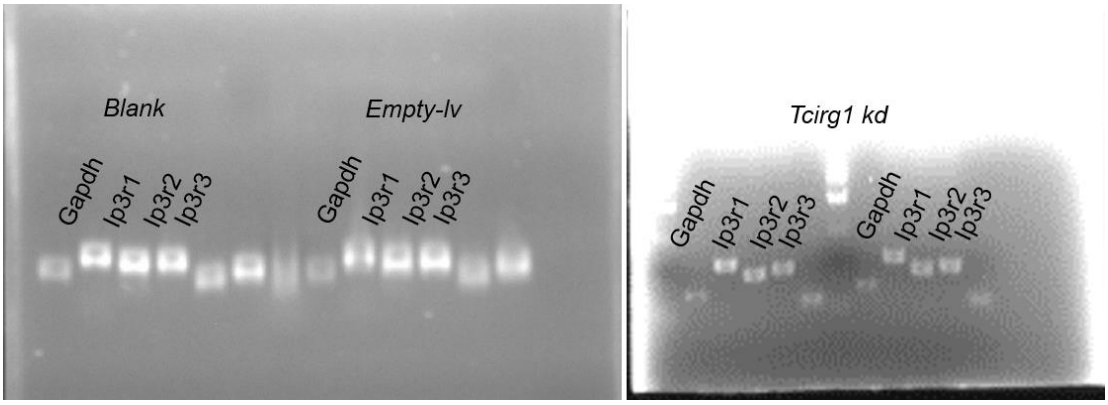

Supplement: S1 Raw images — (PDF) [file pone.0237354.s003.pdf]
